# Imaging of Peritoneal Surface Malignancies

**DOI:** 10.1002/jso.27979

**Published:** 2024-11-07

**Authors:** Damiano Caruso, Paolo Sammartino, Michela Polici, Giorgio Masci, Daniele Biacchi, Marta Zerunian, Daniele Scuto, Maria Gloria Gallotti, Franco Iafrate, Andrea Laghi

**Affiliations:** ^1^ Department of Surgical and Medical Sciences and Translational Medicine Sapienza University of Rome ‐ Sant'Andrea University Hospital Rome Italy; ^2^ Department of Surgery “Pietro Valdoni”, Cytoreductive Surgery and HIPEC Unit Sapienza University of Rome Rome Italy; ^3^ Department of Medical and Surgical Sciences and Translational Medicine, PhD School in Traslational Medicine and Oncology, Faculty of Medicine and Psychology Sapienza University of Rome Rome Italy; ^4^ Department of Radiological, Oncological and Pathological Sciences, Policlinico Umberto I Sapienza Unversity of Rome Rome Italy

**Keywords:** neoplasm metastasis, neoplasms, peritoneum, radiology

## Abstract

Management of peritoneal surface malignancies is currently entrusted to a multimodality approach. Computed tomography (CT) scan remains the first imaging method despite the limitations in identifying small implants in critical regions. Magnetic resonance imaging is usually recommended for its performance in small implants, mesentery, and small bowel assessment. Positron emission tomography/CT plays an important role only in pseudomyxoma peritonei. Thus, becoming aware of the imaging strengths and drawbacks and having a multimodality imaging approach might be the best option for the patients.

## 1 Introduction

Tumors involving the peritoneal cavity, currently defined as peritoneal surface malignancies (PSMs), occur most frequently through the endocavitary spread of gastrointestinal tract neoplasms or the female reproductive system. Still, they may also arise from primary neoplasms of the peritoneum (peritoneal mesothelioma or primary serous papillary carcinoma of the peritoneum) or, more rarely, from extraperitoneal primary tumors [1]. More specifically, in the last three decades, the treatment of PSMs has become increasingly widespread thanks to the adoption, in selected cases, of cytoreduction procedures associated or not with hyperthermic intraperitoneal chemotherapy (HIPEC), techniques introduced in the mid‐1990s in the United States, by the charismatic leader in this field Professor Paul Sugarbaker [2]. Although many questions remain unanswered, the encouraging long‐term results observed and the safety of the procedures used, mainly at high‐volume PSM centers, make this chapter of surgical oncology one of the most innovative and prolific from a scientific point of view, as demonstrated for example, by the 1400 articles published on the subject in 2022 alone [3]. The development of this discipline raises important issues regarding the search for ideal diagnostic tools that can correctly assess the disease balance, track its evolution during neoadjuvant systemic treatments, provide reliable elements for establishing surgical guidelines, and finally, follow‐up resected patients over time to discover recurrences promptly.

At all these stages of the clinical history of PSM patients, two diagnostic methods are available and often wrongly considered alternatives: imaging, which is noninvasive and can ubiquitously assess the spread of disease, and the so‐called “staging laparoscopy,” which allows a direct view of the peritoneal cavity. Staging laparoscopy has specific indications that do not conflict with imaging, as it is mainly used to carry out histological sampling when it is essential to know the origin of peritoneal spread or when a direct vision of the cavity is expected to provide answers, resectability, or recurrence, that images are unable to give with certainty.

In everyday clinical practice, imaging is the most widely used tool in the work‐up of the PSMs patient, and the qualitative and quantitative evaluation of the disease spread must guide the treatment choice. Apart from the standard limitation of the various image techniques related to the drop‐off sensitivity for nodules < 5 mm or the thickening of the peritoneal layers, it is now clear that the specific experience level of the radiology team substantially influences both the choice of the most suitable investigation for the individual patient and the level of sensitivity of the clinical interpretation of the images [4]. The experience of radiological groups with expertise in this field has led to research on the use of increasingly reliable methods such as dual‐energy computed tomography (DECT), spectral photon‐counting CT (SPCCT), or Gallium‐68‐labeled fibroblast activation protein inhibitor (Ga^68^‐FAPI) positron emission tomography combined with CT (PET/CT), and at the same time highlights the need to provide the clinician with a structured report giving information on the number of affected regions and elements that can guide the most suitable therapeutic choice, such as nonresectability criteria [5, 6, 7]. An ideal structured report is thus automatically able to provide a radiological staging of PSMs following the Peritoneal Cancer Index (PCI) initially proposed by Jacquet and Sugarbaker [8], partly due to the inadequacy of the now‐dated classification system [9], partly because of the intrinsic limitations of the technology currently available to us, frequently understage the disease compared to intraoperative findings.

This review aims to take stock of the current state of imaging techniques in the work‐up of different clinical‐pathological aspects of the set of PSMs that are generally treated and, simultaneously, to evaluate the ability of modern imaging technologies to improve staging at the various therapeutic moments.

## 2 Imaging Findings

Imaging is pivotal in evaluating PSMs, providing essential anatomical information, and supporting the oncologist and the surgeon in choosing the best therapeutic approach.

**Figure 1:**
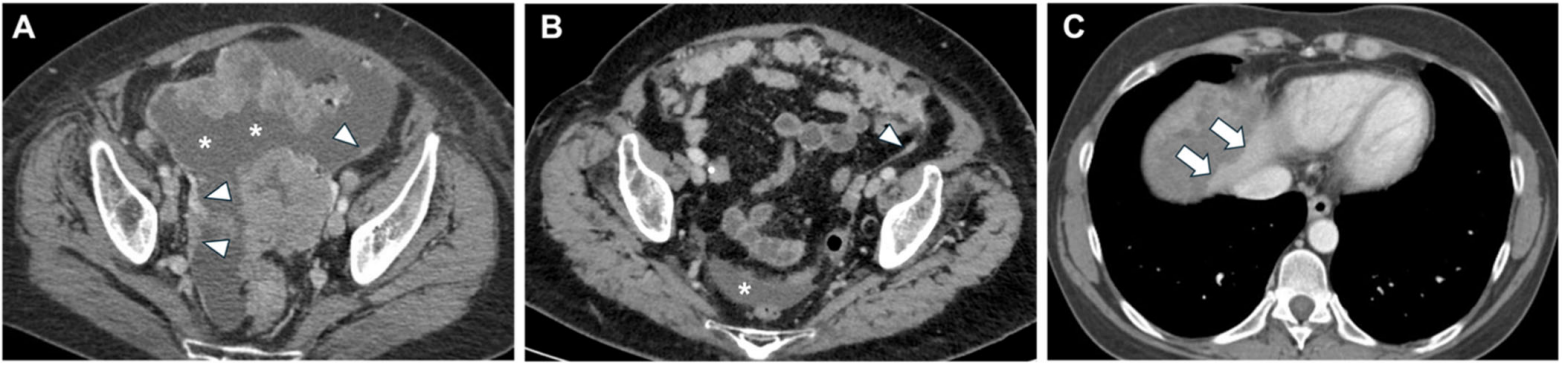
Thickening and enhancement of peritoneal reflections (white arrowheads) with ascites (white star) (A and B). Thickening of right diaphragm (C) with the scalloping of liver surface (white arrows).

**Figure 2:**
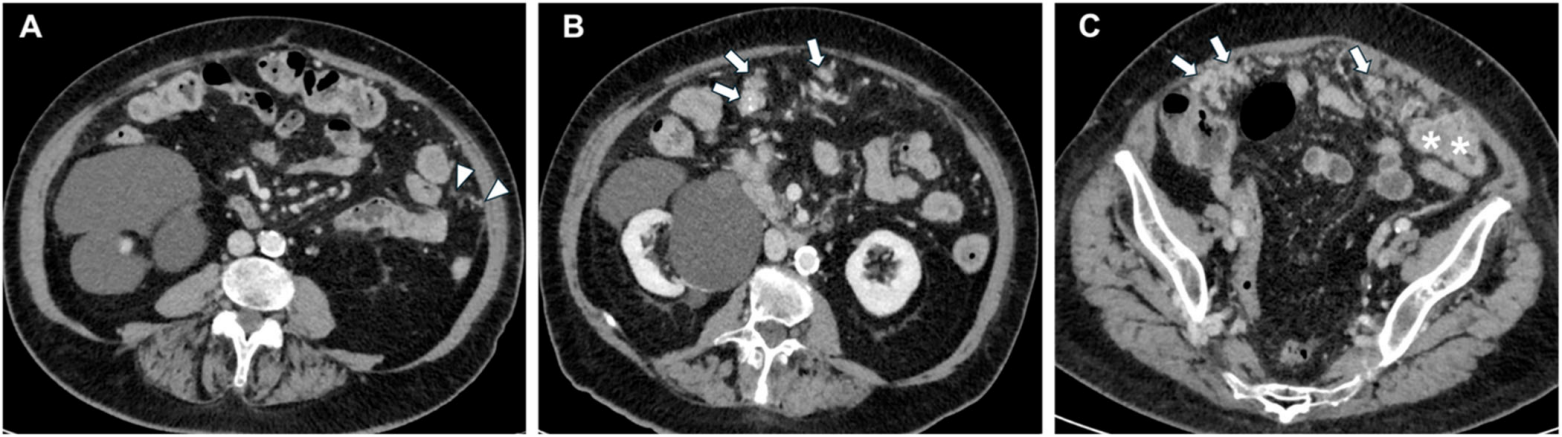
Female, 73‐year old, affected by gastric cancer with peritoneal involvement with micronodular (A, white arrowheads), nodular (B and C, white arrows), and plaque‐like (C, white stars) implants.

**Figure 3:**
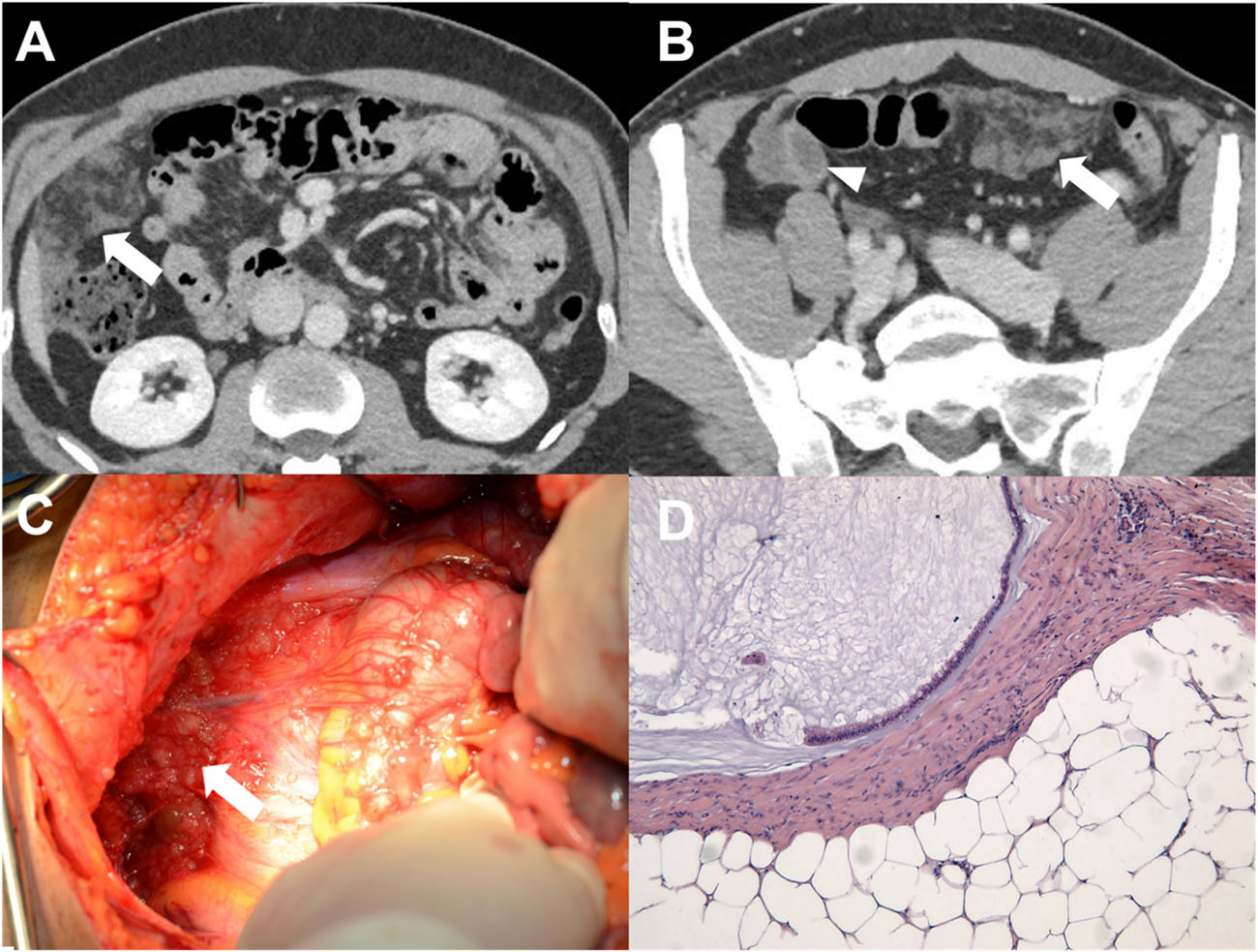
Cystic implants of pseudomyxoma peritonei (arrows) deriving from appendiceal tumor (arrowhead) on computed tomography images (A and B) with intraoperative and histological correlations (C and D).

**Figure 4:**
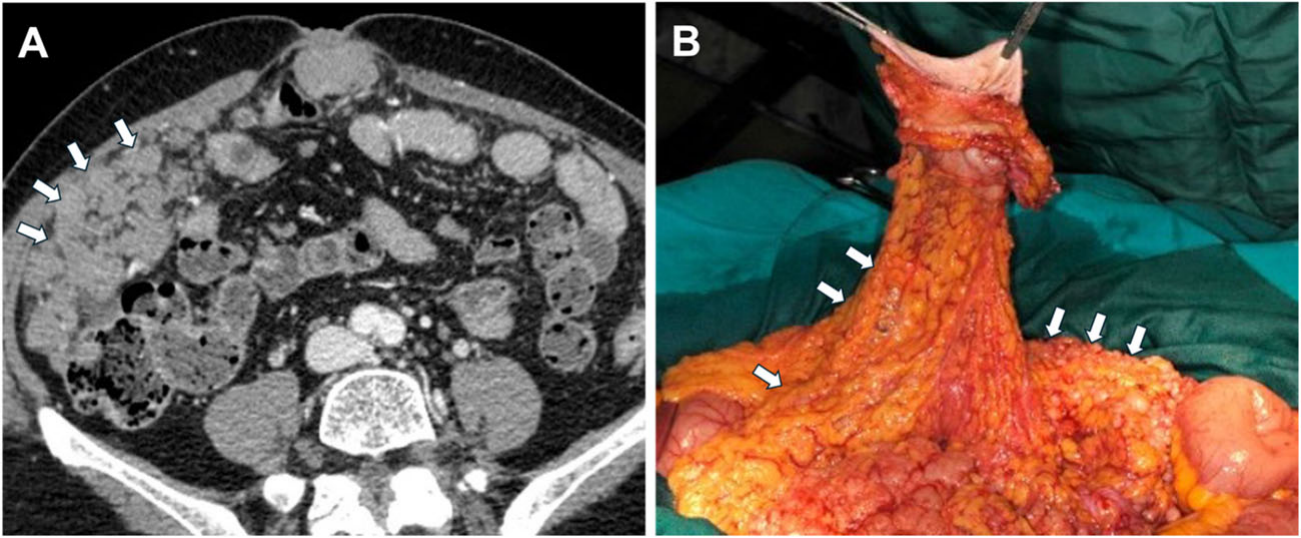
Male, 68‐year old, affected by gastric cancer with stranding and thickening of the omentum (omental cake) (A, white arrowheads), confirmed at surgical inspection (B, white arrowheads).

**Figure 5:**
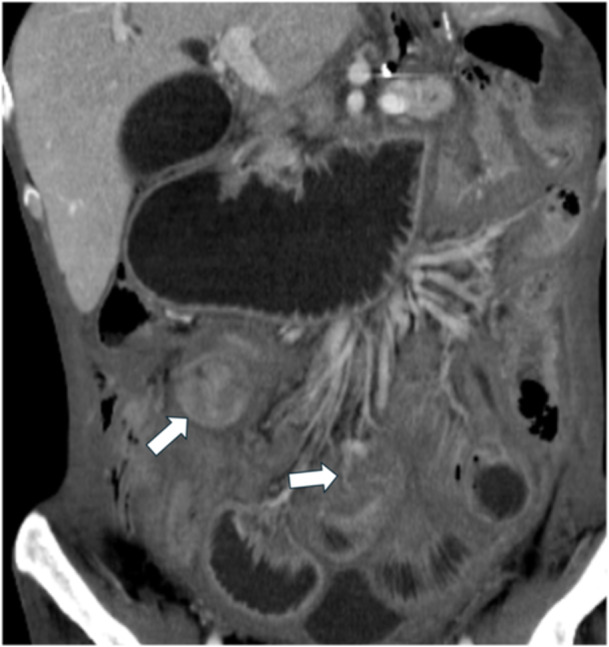
Diffuse thickening of mesentery with involvement of small bowel loop and occlusion (white arrows).

**Figure 6:**
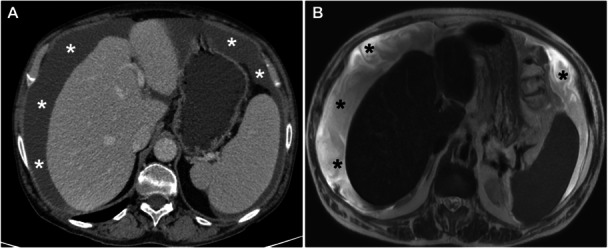
Female, 82‐year old, on computed tomography portal venous phase (A) and magnetic resonance imaging T2w (B), affected by colon cancer with peritoneal carcinomatosis and ascites (white and black stars in A and B, respectively).

**Figure 7:**
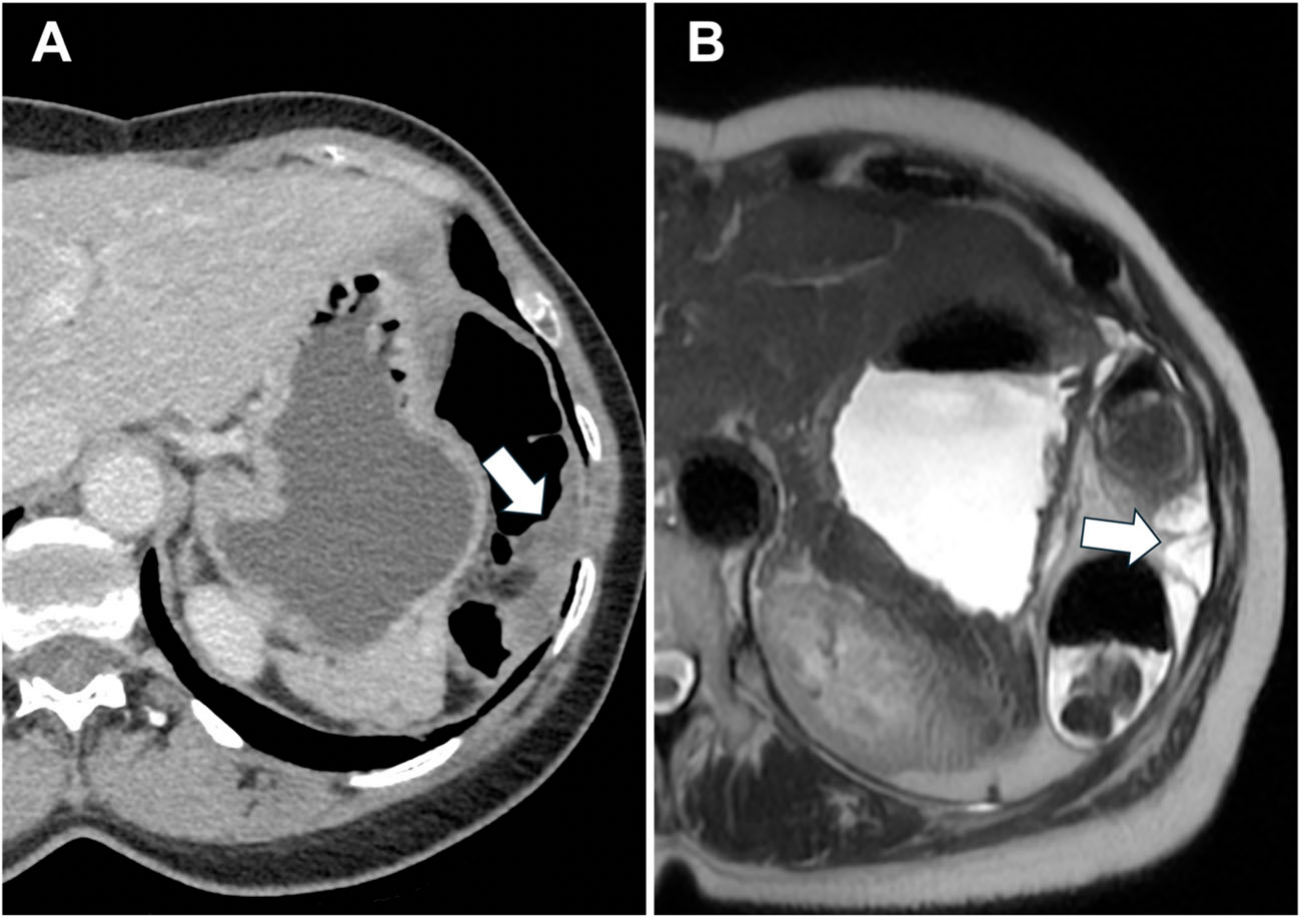
Female, 56‐year old, affected by peritoneal mesothelioma with loculated ascites on computed tomography portal venous phase (A) and magnetic resonance imaging T2w (B) (white arrows).

**Figure 8:**
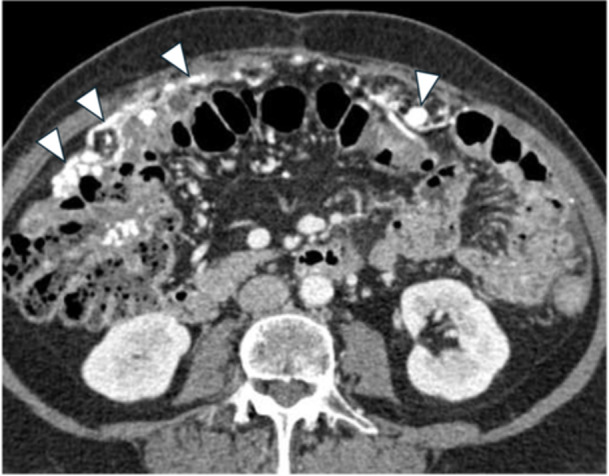
Female, 68‐year old, affected by gastric cancer with calcifications (white arrowheads).

The main imaging findings related to PSMs are the following **(**Table 1):

- Thickening and enhancement of peritoneal reflections, which might be linear thickening or have a nodular appearance, give a plaque‐like appearance in all cases of high disease burden. When encountered along the lower surface of the right or left diaphragm, they may indent the visceral surface of the liver or the spleen, thus mimicking hepatic/splenic metastases, an aspect known as “scalloping” [10] (Figure 1).
- Soft tissue nodules range from micronodular or nodular (1–5 mm and up to 5 cm, respectively) to larger masses resulting from the confluence of multiple nodules (>5 cm). Their size, morphology, and content may be different among PSMs, such as cystic lesions, typical of pseudomyxoma peritonei (PMP) or benign multicystic mesothelioma, and solid contents, primarily prevalent in peritoneal carcinomatosis in gastrointestinal or ovarian cancers [11] (Figures 2 and 3).
- Stranding and thickening of the omentum, known as omental cake, typically occur in gastrointestinal and ovarian carcinomas. The omentum involvement initially appears as increased attenuation of the omental fat with reticulonodular or nodular pattern. Thus, in advanced stages, more extensive tumor deposits might give a thick, soft tissue plaque‐like appearance with posterior dislocation of bowel loops [12] (Figure 4).
- Stranding and distortion of the small bowel mesentery in all cases of tumor infiltration, making the mesentery stiff with loss of its regular undulations and congestion of mesenteric vessels. Perivascular spaces and vessels' infiltration might appear denser than the surrounding fat, referred to as “stellate mesentery,” typically occurring in peritoneal lymphomatosis. The extensive involvement of the mesentery is characterized by diffuse thickening, distortion, and fixation of the mesentery, a condition known as “frozen mesentery,” representing a lethal prognostic factor and possibly leading to small bowel obstruction [13] (Figure 5).
- Ascites are primarily present, which might be an early sign of PSMs. Peritoneal effusion is mainly caused by increased capillary permeability and fluid production or by obstructed lymphatic vessels and decreased absorption (Figure 6). Ascites can be either diffuse or loculated, often associated with PMP or peritoneal mesothelioma, determining scalloping of intraperitoneal organs [14] (Figure 7).
- Calcifications could eventually occur, most commonly in serous ovarian cystadenocarcinoma or gastric carcinoma as the primary site. However, calcifications within nodules may also appear after chemotherapy as a response to therapy. Moreover, a plaque calcification involving the visceral and parietal peritoneum without evidence of significant soft‐tissue mass might be present in well‐differentiated papillary mesothelioma (Figure 8) [15].

**Table 1:** The most common imaging findings of peritoneal surface malignancies.

Findings	Frequency	Histology
Thickening and enhancement of peritoneal reflections	+++	All PSMs
Soft tissue nodules	+++	All PSMs
Cystic implants	+	PMP or PM
Stranding and thickening of the omentum (omental cake)	+	PC
Stranding and distortion of the small bowel mesentery	+	PC and PL
Ascites	+++	All PSMs
Calcifications	+	Mostly PC in ovarian cancer

Abbreviations: PC, peritoneal carcinomatosis; PL, peritoneal lymphomatosis; PM, peritoneal mesothelioma; PMP, pseudomyxoma peritonei; PSMs, peritoneal surface malignancies; +++, highly frequent; ++, common; +, rare.

## 3 Staging and Restaging Criteria

Staging systems in oncology allow the stratification of patients based on the extent of disease and are crucial for patient management both at diagnosis when choosing the best therapeutic option and during follow‐up to assess response after treatment.

**Figure 9:**
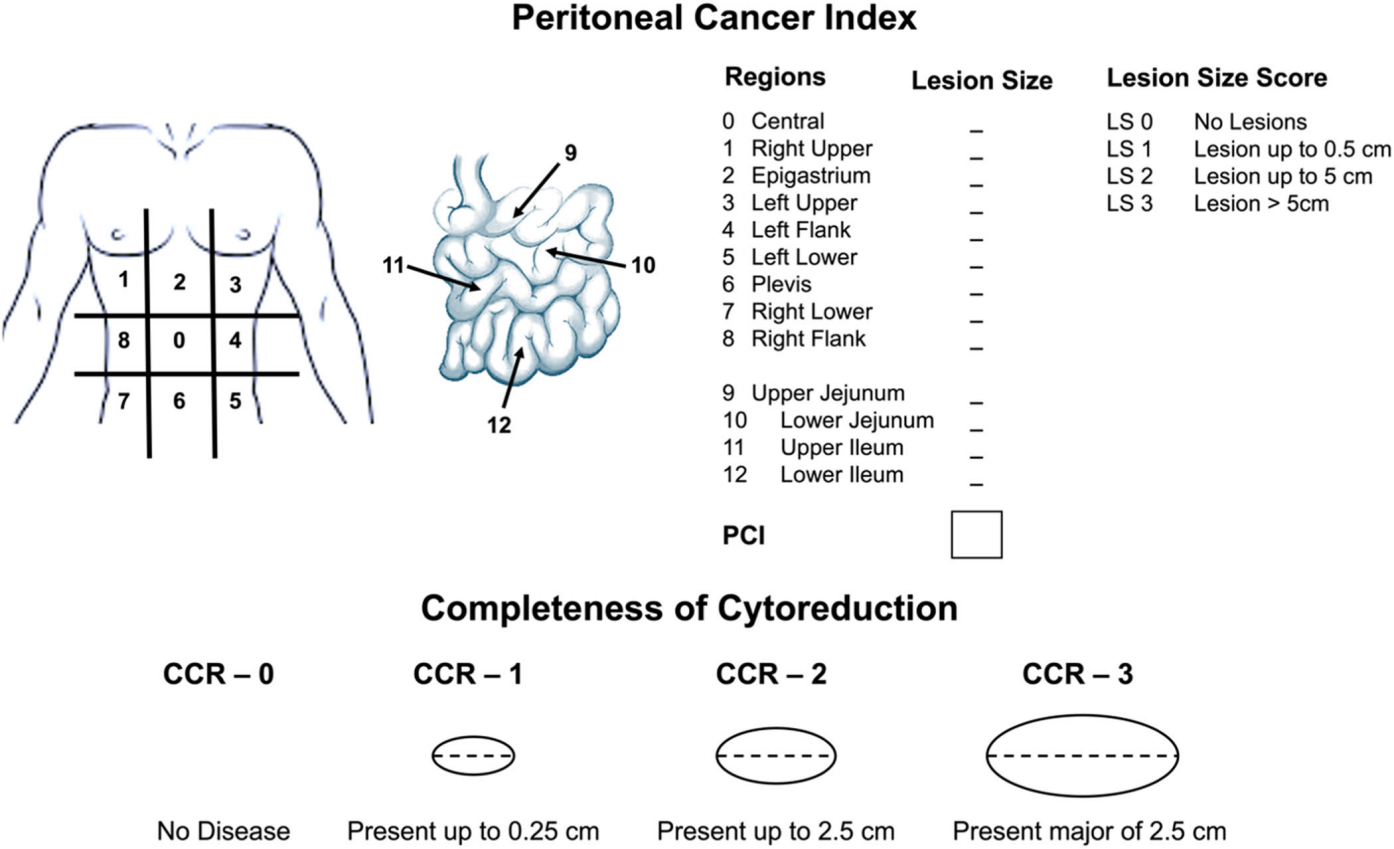
Schematic representation of Peritoneal Cancer Index and Completeness of Cytoreduction score.

To date, the staging and re‐staging of PSMs consists of two main approaches developed by Sugarbaker: the PCI, and the completeness of cytoreduction score (CCR) [8]. The PCI is the most used; it is a surgical score calculated by dividing the abdomen into 13 regions and measuring the size of the most prominent implant (0–3) in each area for a maximum score of 39 (Figure 9). In each region, the largest diameter of the implants is measured and graded as follows: 0 points for no visible tumor, 1 point for lesions < 0.5 cm in diameter, 2 points for lesions between 0.5 and 5 cm, and 3 points for lesions > 5 cm. The same scoring system can be applied to imaging instead of CT and MRI, providing a preoperative assessment of tumor burden. PCI is the most validated score of patient prognosis; when PCI is 20 or greater, the chances of obtaining a complete cytoreduction are meager. Jejunal regions 9 and 10 are also associated with unfavorable prognoses compared to ileal regions 11 and 12 [16]. Including the PCI in the radiological report may be fundamental for surgical planning, facilitating communication between the radiologist and surgeon [17].

The benefit of using PCI goes beyond the description of the extent of peritoneal tumor involvement. It also plays a vital role in treatment decisions, differing from one histotype to another. For instance, while patients with PMP may benefit from the combination of cytoreductive surgery (CRS) and HIPEC even with high PCI, the same multimodal therapy should be limited to a lesser degree of peritoneal involvement (PCI < 6) in patients with gastric carcinoma [18].

Looking at re‐staging evaluation after cytoreduction, it is fundamental to consider the CCR, aiming to assess the residual disease on a 4‐point scale (CCR‐0 to CCR‐3) based on size. More specifically, CCR‐0 is achieved when there is no residual macroscopic tumor, CCR 1 when residual tumor deposits are < 2.5 mm in diameter, CCR‐2 when residual tumor deposits range from 2.5 mm to 2.5 cm, and CCR‐3 indicates nodules > 2.5 cm (Figure 9) [8]. Assessment of CCR is crucial for addressing patients to further treatment, such as the HIPEC, generally indicated in patients with CCR‐0 and CCR‐1 [9]. Thus, PCI and CCR are the two most critical quantitative scores that can predict patient prognosis and could be used by surgeons to identify patients who could benefit from aggressive and invasive surgery.

In addition to PCI, the CCR is a surgical score, and its applicability to imaging could be seen as a real challenge. While the literature data concerning imaging performance in predicting PCI are highly available, only a few pieces of evidence tested the role of CT and MRI in predicting CCR. Still, the emerging results demonstrated that CT could not be accurate, while MRI might be promising and more effective than CT [19]. Similarly, MRI‐DWI might help predict complete cytoreduction before the surgery, avoiding invasive surgery in patients who could not benefit [20]. However, no recommended guidelines are available to standardize the assessment of CCR in a preoperative setting.

Nevertheless, PCI and CCR scores have consistent drawbacks: the two‐dimensional nature, manual performance, and high time consumption, and in the same region, several organs overlap. Thus, to facilitate surgical planning and to overcome these aspects, Sammartino P. and colleagues proposed a computerized version for PSMs staging and re‐staging with more accurate information and a three‐dimensional illustration of the peritoneal cavity, allowing a more comprehensive staging system [9]. They maintained the cornerstones of PCI and CCR, proponing small changes: assigning to each group of organs a region (e.g., the omentum matches the area 0) to overcome the structures' overlap, differentiating the anatomy between male and female, providing a three‐dimensionality, structuring the information to make more accessible the language between radiologists and surgeons, and a friendly web abscess established. This could be understood as the most relevant attempt to propose a structured report with free access. To our knowledge, there is no literature on structured reports in PSMs. However, the need for the introduction of structured reports in cancer patients has recently been emphasized, both for universal multidisciplinary communication and to create online databases that can be queried through the natural language processing approach, thus being usable for research purposes in a structured manner [21].

## 4 Imaging Modalities: Current Evidence

**Figure 10:**
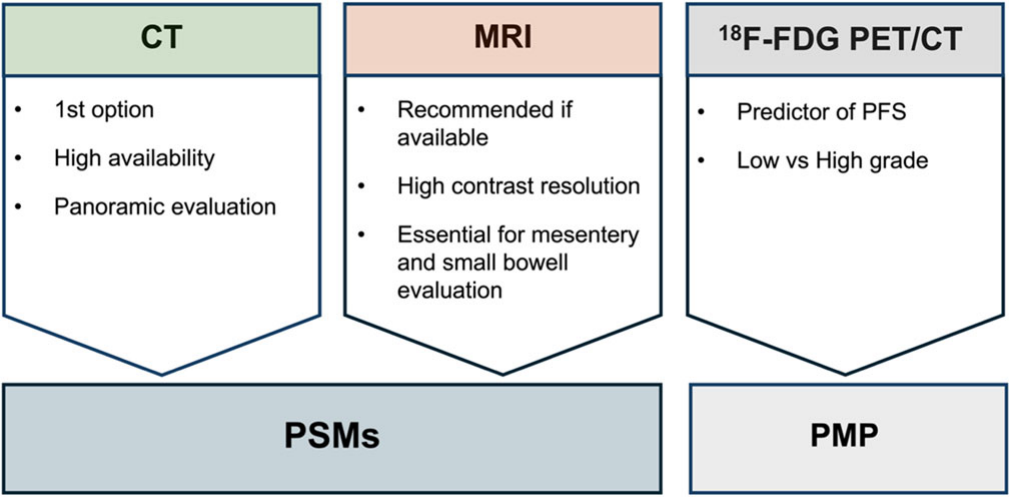
Imaging modalities in preoperative setting: Current evidence. CT, computed tomography; ^18^F‐FDG; 18 F‐2‐deoxy‐2‐fluro‐d‐glucose; MRI, magnetic resonance imaging; PET, positron emission tomography; PMP, pseudomyxoma peritonei; PSMs, peritoneal surface malignancies.

CT scans are the first imaging option to assess peritoneal malignancies and plan the surgery due to high availability, cost‐effectiveness ratio, and spatial resolution (Figure 10) [22, 23]. Its accuracy mainly depends on the maximum diameter and location of peritoneal implants and the presence of ascites, which could increase CT performance [24, 25, 26]. CT has a deficient performance in the evaluation of small peritoneal implants ( < 1 cm), in which the literature data showed a sensitivity ranging from 9.1% to 50%, setting the lowest cut‐offs for lesion detectability at 5 mm with a sensitivity around 11% [23]. Beyond the dimensional limitation, the localization of small peritoneal lesions, such as in the small intestine mesentery, right upper abdomen, and lower abdomen should be considered [22, 23]. In surgical planning, specific information about general PCI and the eventual involvement of particular anatomical regions, such as hepatic hilum or small intestine (region 2 + 9 to 12), is essential, which could compromise the surgical outcome. Looking at the assessment of general CT‐PCI, some intermediate results were achieved with a sensitivity of 76% and specificity of 69% [27].

In such a scenario, MRI gets the role of an alternative imaging method, understood as a problem solver or an adding tool to improve imaging performance in small implants or involvement of specific anatomical regions, such as the small intestine (Figure 10). In the last few years, diffusion‐weighted imaging (DWI) has reached an increasing role due to its good sensitivity in detecting small lesions, especially in specific abdomen regions and in dedicated histology (e.g., PMP or mucinous) [22, 28, 29]. Moreover, MRI‐DWI seems to improve the detection of malignant deposits in locations crucial for determining patients' eligibility for surgery, such as the mesenteric root, Treitz ligament, small bowel wall, and hepatic hilum [30]. In the head‐to‐head comparisons between imaging and laparoscopy, MRI showed higher sensitivity than CT in detecting small lesions. Still, no statistically significant results were achieved regarding the imaging performance [20, 31, 32]. However, a substantial limitation of MRI is the lack of concordance in the interobserver agreement; it ranges from excellent (*k* = 0.92) to intermediate results (*k* = 0.58) and is usually not significantly superior to CT scan (*p* > 0.05) and is primarily dependent on lesion diameter and histology [20, 28, 33]. This datum depends mainly on the reader's experience, and the need for extensive studies, including comparisons between CT, MRI, and laparoscopy, limits the use of MRI. Moreover, MRI performance is usually reduced in some specific regions in the upper abdomen, such as the diaphragm and pericardiophrenic areas, due to cardiac pulsatility and air‐surface artifacts [34].

PET‐CT represents a highly accurate and extensively used technique in the oncological setting, allowing the detection of tissues with pathological uptake of the radiotracer, most commonly 18 F‐2‐deoxy‐2‐fluro‐d‐glucose (^18^F‐FDG PET/CT). The role of ^18^F‐FDG PET/CT is still limited PSMs due to the physiological uptake of several anatomic structures, including the liver capsule, small bowel, or kidney, and spontaneous uptake in almost all cases of infections [35, 36], and the potential false‐negative results, related to mucinous tumors (e.g., deriving from the appendix, colon, or ovary), signet‐ring gastric cancers, and small tumor deposits [10]. A recent meta‐analysis might place ^18^F‐FDG PET/CT on the same level as MRI, outperforming CT scan; a careful analysis reveals that the number of studies included is unbalanced in favor of the CT scan [22]. Then, an intrinsic selection bias could lead to partial and misleading conclusions. Recently, PET/MRI has been tested as an alternative to standard of care, understood as contrast‐enhanced CT, ^18^F‐FDG PET/CT, or contrast‐enhanced MRI, and some promising results were achieved showing a PET/MRI superiority in terms of sensitivity and comparable results of overall specificity [37]. This data was confirmed in a similar study, in which PET/MRI accurately assessed per‐patient radiological PCI (85.7%). Still, the per‐region radiological PCI remains poor (66.5%) [38].

One of the few utility fields of ^18^F‐FDG PET/CT is PMP, which differentiates low‐ from high‐grade, with an area under the curve around 70%, and predicts patient outcome regarding progression‐free survival by setting a PET‐PCI at 12 [39, 40]. However, the international consensus on the applicability of PET/CT in the preoperative evaluation of PMP still needs to be improved.

## 5 One Diagnostic Test Does Not Fit All: A Personalized Diagnostic Strategy

Overall, diagnostic imaging plays a critical role in differentiating candidates for CRS from patients with extensive peritoneal disease or unfavorable lesion sites who should undergo neoadjuvant or palliative chemotherapy [41, 42]. Preoperative imaging is, therefore, essential to guiding surgeons in treatment planning, helping to define tumor burden, thus providing information on resectability and predicting survival outcomes. Moreover, the radiological challenge is to choose the best imaging option to assess the PCI in a preoperative setting without a literature consensus. As well as setting any imaging PCI cut‐offs in a preoperative clinical setting is one of the most critical unmet surgical needs. From a surgical perspective, some recent studies proposed different PCI cutoffs to ensure complete resectability. Although there are no universal criteria for inoperability, and decisions are usually made based on the experience of each center, three main criteria are considered for eligibility for surgery: the extent of peritoneal disease, the possibility of obtaining complete cytoreduction, and postoperative quality of life [41, 42]. Causes of nonresectability include the presence of extraperitoneal metastases, retroperitoneal lymphadenopathies, diffuse peritoneal involvement (conventionally PCI > 10), or the participation of specific abdominal sites, such as the digestive tract, the hepatic pedicle or hilum, the mesenteric root, and central pelvic involvement (mainly the bladder) [42, 43]. More specifically, a PCI cutoff has been proposed for peritoneal carcinomatosis in ovarian cancer to predict complete cytoreduction after neoadjuvant chemotherapy, which was set under 17, showing the best predictor for the surgical outcome [44]. Looking at the best imaging option, in most PSMs can be followed the aforementioned data concerning the conventional CT as the first option and possibly complemented by MRI to try to bridge the gaps in the two methods, providing the surgeon with as much information as possible, always keeping in mind the existence of a mismatch between radiologist and surgeon, and continuing to consider the PET/CT as a secondary option due to the high level of biases [20, 31, 32, 35, 36].

Conversely, in patients affected by PMP, it is necessary to stratify the patients into low‐ and high‐grade tumors with different prognoses and then set different general PCI cutoffs at 21 and 25 for resectability, respectively [45]. Therefore, a precise radiological report that addresses findings that potentially preclude CRS allows for the reduction of unnecessary surgery and associated mortality. Alternative treatments, such as neoadjuvant or palliative chemotherapy, may be considered for patients eligible for CRS and HIPEC. While from a surgical point of view, PCI cut‐offs are a real option, from a radiological point of view, there is still a long way to go, as demonstrated in a recent study in which setting a CT‐PCI cutoff at 20 was proved to be inaccurate, considering that almost half of the patients were still eligible for complete cytoreduction even if radiologically classified as unresectable [27]. Thus, the new emerging data suggested the identification of non‐resectable patients according to the involvement of regions 2 + 9 to 12. Still, most studies showed a need for more reliability in the case of small implants [46, 47]. These conflicting data arise from the chose of imaging method for PMP assessment, while CT remains the first option, emerging data demonstrated that MRI may be superior in identifying small lesions in small bowel serosa and in the mesentery involvement (sens: 67 vs. 88% and spec: both 100% in CT vs. MRI, respectively) [28]. Reflecting the need to integrate CT and MRI also in the preoperative evaluation of PMP. But in the assessment of PMP, we cannot stop at CT and MRI evaluation alone, in fact, a predominant role is currently played by the ^18^F‐FDG PET/CT, which is able to predict both the risk of recurrence and tumor grading [48, 49]. More specifically, it has been recognized that the maximum standardized uptake value (SUVmax) might be an independent predictor factor of progression‐free survival with an SUVmax cutoff established at 2.02 on preoperative ^18^F‐FDG PET/CT (Figure 10) [48]. Moreover, the SUVmax might be used to predict the tumor grading on a preoperative clinical setting, achieving good sensitivity and specificity (77.8% and 72.3%, respectively) setting cutoff of 2.63, however, in the same study was also investigated the comparison with CT, which resulted to be superior than ^18^F‐FDG PET/CT in small implants detectable as small anatomical irregularities [49]. These data highlighted the role of metabolic imaging as predictor of prognosis, helpful to select patients with resectable disease, but conventional CT remains to be highly recommended PMP assessment.

## 6 Future Perspectives

Considering the several limitations of conventional imaging, including CT, MRI, and PET/CT, the new landscape of imaging includes the use of DECT, with the chance to improve the contrast resolution and SPCCT, able to rise the spatial resolution to 250 µm [50]. Recently, DECT was demonstrated to be superior to conventional CT scans to diagnose occult peritoneal metastases in advanced gastric cancer, with the possibility to avoid any unnecessary laparoscopy and tailor neoadjuvant chemotherapy without any invasive diagnostic approach [51]. While only a few data were published in the literature about the applicability of SPCCT, the general trend moves toward the higher special resolutions due to the lack of septa between the detectors, translating into higher accuracy and sensitivity [5, 52].

From a nuclear medicine perspective, [^68^Ga]Ga‐FAPI PET/CT has been acquiring a relevant role in peritoneal malignancies. The FAPI is a new radiopharmaceutical labeled with either 68Ga or 18F, targeting the fibroblast activation protein, usually overexpressed in cancer‐associated fibroblasts (CAFs) and rarely expressed in normal tissues [53]. CAFs are part of the peritumoral stroma in many epithelial tumors and contribute significantly to tumor mass; notably, tumors larger than 2 mm have a support matrix larger than the tumor volume [54, 55]. Then, the hypothesis is that a radiopharmaceutical targeting the peritumoral stroma could detect small micronodular carcinosis lesions more sensitive than ^18^FDG‐PET/CT [56, 57]. Some recent results demonstrated the strengths of [^68^Ga]Ga‐FAPI PET/CT compared to ^18^FDG‐PET/CT in terms of sensitivity in both per patient (98.2% vs. 55.9%) and per‐lesion (99.9% vs. 27.3%), particularly the specificity reached values around 100% [58]. Moreover, [^68^Ga]Ga‐FAPI PET/CT demonstrated a higher detection rate in peritoneal metastases than ^18^FDG‐PET/CT in a recent meta‐analysis [59].

Overall, the new imaging methods described demonstrated promising results. They should be validated in extensive multi‐center studies to become safe and reliable in clinical settings. Further developments are expected by the application of AI‐based software which might be helpful in assessing volumetric tumor burden and categorizing tumor response to therapy.

## 7 Conclusions

PSMs are a heterogeneous group of diseases, in which applying a rigid imaging protocol might be reductive, but the individualized approach per pathology can be the winning weapon. Integration of the various imaging methods (CT, MRI, and ^18^FDG‐PET/CT) should always be considered to minimize the inherent bias of each method. In the future new imaging methods, such as DECT, SPCCT, and [^68^Ga]Ga‐FAPI PET/CT, might be included in PSMs management. In the end, a collaboration between radiologists and surgeons is necessary to select patients eligible for surgery and to tailor per‐patients the best therapeutic options.

## Synopsis

CT is currently considered the first imaging method; MRI might be superior to CT in small lesions and dedicated abdominal regions. PET/CT has a role only in pseudomyxoma peritonei. Thus, a multimodal imaging approach could always be considered in malignant peritoneal surface tumors to obtain the best treatment options for patients.

## Data Availability

Data sharing is not applicable to this article as no new data were created or analyzed in this study.
